# American Naturalist

**Published:** 1867-06

**Authors:** 


### *American Naturalist.*

We welcome to our exchange list this beautifully printed and illustrated, and ably edited monthly. It is published by an association of naturalists, at Salem, Mass., and afforded at the nominal price of $3.00 *per annum*. All lovers of natural history should subscribe for it.

